# Changes in Mood and Tryptophan Metabolism Following a 12-Week Endurance Exercise Combined with Time-Restricted Eating in Healthy Adults

**DOI:** 10.3390/nu18193116

**Published:** 2026-09-22

**Authors:** Ulana Juhas, Dagmara Budnik-Przybylska, Andżelika Borkowska, Jakub Antoni Kortas, Joanna Reczkowicz, Ewa Ziemann, Katarzyna Prusik, Damian Flis, Grażyna Gałęzowska, Konrad Kowalski, Malgorzata Zychowska, Barbara Kaczorowska-Hać, Jędrzej Antosiewicz

**Affiliations:** 1 Department of Bioenergetics and Physiology of Exercise, Medical University of Gdansk, Dębinki 1, 80-211 Gdansk, Poland; andzelika.borkowska@gumed.edu.pl (A.B.); joanna.reczkowicz@gumed.edu.pl (J.R.); konrad.kowalski@masdiag.pl (K.K.); jedrzej.antosiewicz@gumed.edu.pl (J.A.); 2 Division of Sport Psychology, Faculty of Social Sciences, Institute of Psychology, University of Gdansk, Bazynskiego 4, 80-309 Gdansk, Poland; dagmara.budnik-przybylska@ug.edu.pl; 3 Department of Health and Natural Sciences, Gdansk University of Physical Education and Sport, Kazimierza Gorskiego 1, 80-336 Gdansk, Poland; jakub.kortas@awf.gda.pl; 4 Department of Athletics, Strength and Conditioning, Poznan University of Physical Education, Krolowej Jadwigi 27/3, 61-871 Poznan, Poland; ziemann@awf.poznan.pl; 5 Department of Sport, Gdansk University of Physical Education and Sport, Kazimierza Gorskiego 1, 80-336 Gdansk, Poland; katarzyna.prusik@awf.gda.pl; 6 Department of Pharmaceutical Pathophysiology, Faculty of Pharmacy, Medical University of Gdansk, 80-210 Gdansk, Poland; damian.flis@gumed.edu.pl; 7 Department of Environmental Engineering Technology, Faculty of Civil and Environmental Engineering, Gdansk University of Technology, Narutowicza Str. 11/12, 80-233 Gdansk, Poland; 8 Department of Sport, Faculty of Physical Education, Kazimierz Wielki University, 85-064 Bydgoszcz, Poland; malgorzata.zychowska@ukw.edu.pl; 9 Faculty of Health, WSB Merito University, 80-266 Gdansk, Poland; barbara.kaczorowska-hac@gdansk.merito.pl

**Keywords:** kynurenines, tryptophan metabolism, anxiety, well-being, inflammation

## Abstract

**Background**: The interplay between time-restricted eating (TRE), endurance exercise, mood, and tryptophan (TRP) metabolism has not been thoroughly examined. This study evaluated whether changes in psychological well-being co-occurred with changes in serum kynurenine metabolites during a combined TRE and exercise intervention and performed exploratory analyses of kynurenine metabolism. **Methods**: Seventy-one healthy adults participated in a 12-week endurance training program combined with a 10 h TRE window. Assessments included the State-Trait Anxiety Inventory, Mood Adjective Checklist, body composition, inflammatory markers, and serum kynurenine profiling, conducted at baseline and post-intervention. The pre-specified analysis was performed in the entire cohort, whereas subsequent analyses stratified by age and changes in anxiety were conducted post hoc and should be considered exploratory. **Results**: The pre-specified analysis of the entire cohort did not reveal statistically significant or clinically meaningful changes in psychological well-being, kynurenine metabolites, or inflammatory biomarkers. In exploratory post hoc analyses, participants were stratified into older (OE-TRE) and younger (YE-TRE) groups. Both groups showed significantly lower body mass and BMI at the second measurement. In the OE-TRE group, serum anthranilic acid (AA) levels increased (Δ = +7.85 nmol/L), while in the YE-TRE group, a decrease was observed (Δ = −2.62 nmol/L). In a separate exploratory post hoc analysis, participants with complete psychological assessment data were stratified according to the direction of change in anxiety scores. Among these participants, 66.7% showed lower anxiety scores in the second measurement, and this anxiety-defined subgroup also showed lower serum 3-hydroxykynurenine (3-HK) concentrations. This observation was confined to the exploratory subgroup analysis and should not be interpreted as a general reduction in 3-HK in the study cohort. The OE-TRE group also showed an increase in the xanthurenic acid (XANA) + picolinic acid (PA) to 3-HK ratio and a reduction in TNF-α. Notably, differences between groups in the 3-hydroxyanthranilic acid (3-HAA)/3-HK and XANA + PA/3-HK ratios observed at baseline were no longer present at the second measurement. In the exploratory subgroup of participants whose 3-HAA concentrations increased over time, soluble gp130 (sgp130) concentrations were lower at the second measurement. **Conclusions**: The pre-specified whole-cohort analysis did not demonstrate statistically significant or clinically meaningful changes in the primary psychological and biochemical outcomes. Exploratory post hoc analyses suggest that changes in psychological well-being and peripheral kynurenine metabolism may co-occur during a combined TRE and endurance exercise intervention. The observed decrease in 3-HK was restricted to an anxiety-defined exploratory subgroup and was not observed in the study cohort as a whole. These findings are associative and hypothesis-generating and do not establish an intervention effect or causality. The trial was registered as a clinical trial (NCT05229835, date of first registration: 8 February 2022).

## 1. Introduction

Exercise is one of the most effective lifestyle interventions for improving energy metabolism and mental health [1,2]. Its metabolic adaptations have therefore been extensively investigated, highlighting the complex interplay between physical activity, nutrition and health [3,4]. More recently, growing attention has been directed toward not only diet quality but also the timing of food intake, as an important determinant of metabolic and mental health outcomes [5,6]. One increasingly studied dietary approach is time-restricted eating (TRE), in which food intake is confined to an 8–10 h daytime window, resulting in a daily fasting period of at least 14 h [7].

TRE has also been increasingly studied in combination with regular exercise, with growing interest in the metabolic and psychological changes accompanying such combined lifestyle interventions. Metabolic adaptations observed in studies combining TRE and exercise may involve pathways relevant to central nervous system function, although the underlying mechanisms remain incompletely understood. Physical activity is accompanied by the release of various proteins and metabolites from skeletal muscle, bone, and other tissues, which may contribute to systemic metabolic responses [8,9,10]. The underlying neurobiological mechanisms remain incompletely understood. Of particular interest are kynurenine pathway metabolites derived from tryptophan (TRP), which play regulatory roles in metabolism, immune response, and brain function, including neurodegeneration and neuroprotection [11,12,13]. TRP metabolites circulating in human blood can be broadly divided into those that cross the blood–brain barrier—kynurenine (KYN), 3-hydroxykynurenine (3-HK), picolinic acid (PA), anthranilic acid (AA), and xanthurenic acid (XANA)—and those with limited permeability, such as quinolinic acid (QUIN), kynurenic acid (KYNA), and 3-hydroxyanthranilic acid (3-HAA) [14]. Among them, PA, XANA, and AA have been associated with neuroprotective properties in experimental studies, whereas KYN and 3-HK have been associated with potential neurotoxic effects under certain conditions in previous experimental studies [15].

Because circulating TRP metabolites that can cross the blood–brain barrier may also influence their central levels, their serum concentrations are of particular relevance [14]. In contrast, less permeable metabolites are believed to exert their effects primarily in peripheral tissues. For example, stress-induced upregulation of KYN may enhance pro-inflammatory signaling in the brain, contributing to depressive symptoms [9]. Indeed, elevated KYN and 3-HK levels have been observed in individuals with depression and other mental disorders [15]. Similarly, QUIN has been implicated as a pro-inflammatory and pro-oxidative agent in peripheral tissues [16].

Pro-inflammatory cytokines, including IL-6 and TNF-α, can cross the blood–brain barrier and may contribute to depression when immune signaling persists [17]. IL-6 further enhances IDO activity, the enzyme promoting TRP degradation along the kynurenine pathway and increasing metabolites such as 3-HK and QUIN, which have been associated with pro-oxidant and potentially neuroactive properties under specific experimental or pathological conditions [18,19]. Dysregulation of IL-6 signaling components, such as soluble interleukin-6 receptor (sIL-6Rα) and soluble glycoprotein 130 (sgp130), has also been observed in patients with depression, with sgp130 proposed as a selective inhibitor of IL-6 trans-signaling [20,21]. The KYN/TRP ratio—an indirect measure of IDO1 activity—has been positively associated with systemic inflammatory markers, including TNF-α and others [22].

Tryptophan metabolism is influenced by numerous factors, including age, physical activity, and diet [23,24]. Several studies have shown that serum kynurenine levels differ between younger and older adults [25]. Previous experimental studies have suggested that physical exercise may modulate TRP metabolism by upregulating kynurenine aminotransferases (KATs) in skeletal muscle, thereby promoting the peripheral conversion of KYN into KYNA and altering the circulating kynurenine metabolite profile [26]. Despite growing interest in combining exercise with TRE, little is known about whether this intervention is accompanied by coordinated changes in psychological well-being and circulating kynurenine metabolites, particularly across different age groups. The present study aimed to determine whether changes in psychological well-being following a 12-week intervention combining TRE and regular physical activity co-occur with changes in circulating kynurenine pathway metabolites. We hypothesized that changes in psychological well-being would be accompanied by coordinated changes in circulating kynurenine metabolites. Mental health, assessed using validated psychometric instruments evaluating mood and anxiety, constituted the pre-specified primary clinical outcome. Serum kynurenine metabolites were predefined as the principal biochemical outcome measures, whereas inflammatory markers, including sIL-6R, TNF-α, and sgp130, were evaluated as secondary biological markers. The (XANA + PA)/3-HK ratio was selected a priori exclusively for sample-size estimation because it demonstrated the largest pre–post standardized difference in our pilot dataset; however, it was not designated as a primary study endpoint and was not analyzed as a separate confirmatory outcome. Because baseline kynurenine metabolism differs between younger and older adults [25], age-related differences in changes over time were additionally explored post hoc.

## 2. Materials and Methods

### 2.1. Ethics

All participants were informed about the goals, methods and possible risks related to this study and signed written consent. Ethical approval was obtained from the Committee for Ethics in Scientific Research of the Medical University of Gdansk (Poland; NKBBN/330/2021), in accordance with the Declaration of Helsinki–Ethical Principles for Medical Research Involving Human Subjects. This study was registered as Clinical Trials Effect of Time-restricted Eating and Endurance Training on Markers of the Health of Women and Men (NCT05229835, date of first registration: 8 February 2022, direct link: https://classic.clinicaltrials.gov/ct2/show/NCT05229835). URL (accessed on 8 February 2022). Data files, including personal information, were encrypted. The password is available only to researchers involved in the project. The hard drives are stored in the safe.

### 2.2. Study Samples

The (PA + XANA)/3-HK ratio was selected as the main determinant for the a priori sample size calculation. Using G*Power software (version 3.1.9.7), the required sample size was calculated for a two-group repeated-measures design with two measurement points (within–between interaction). The calculation was based on an expected effect size of f = 0.25, corresponding to a partial eta-squared value of 0.06, a significance level of α = 0.05, power (1 − β) = 0.80, and a correlation between repeated measures of r = 0.60. The power analysis indicated that a total of 28 participants were required to achieve sufficient statistical power, corresponding to a minimum of 14 participants per group.

Healthy volunteers aged ≥35 years were recruited from local senior citizen clubs, third-age universities, church communities, and among employees of the Medical University. All participants were independent in their daily activities and had no severe cognitive or physical impairments. Participant recruitment, eligibility assessment, and study flow are presented in Figure 1. Exclusion criteria included smoking tobacco products, the use of potent stimulants or psychoactive substances, diagnosed neurological disorders, a history of cardiac arrhythmia or unstable cardiovascular disease, dementia, autoimmune diseases, neoplastic conditions, anemia, diastolic blood pressure > 100 mmHg, and chronic conditions requiring pharmacological treatment (e.g., hypertension, diabetes, or active cancer), as these conditions or their treatment could interfere with the interpretation of metabolic and inflammatory outcomes. Participants were excluded from the final analysis because of insufficient adherence to the exercise program or TRE protocol, changes in health status, lack of time, voluntary withdrawal, or loss to follow-up. All participants included in the present analysis received a combined intervention consisting of time-restricted eating (TRE) and an age-appropriate endurance exercise program.

The primary pre-specified analyses comprised within-group pre–post comparisons for the entire cohort, including psychological outcomes, kynurenine metabolites, inflammatory markers, and anthropometric parameters. All subgroup analyses described below were performed post hoc and should be interpreted as exploratory.

Age stratification was not part of the original analytical plan and was introduced post hoc for exploratory purposes to investigate whether age-related differences in kynurenine metabolism observed in previous studies might also be present in the current cohort. This analysis was not pre-specified in the study protocol and should therefore be interpreted as exploratory and hypothesis-generating. The analysis based on the direction of change in anxiety levels was also conducted as an exploratory post hoc analysis because previous studies suggested that anxiety may be associated with alterations in kynurenine metabolism.

### 2.3. Measurement of Body Composition

Body composition was assessed using direct segmental multi-frequency bioelectrical impedance analysis. Body mass was determined after overnight fasting (minimum 12 h). The impedance of body parts (trunk, arm, and leg segments) was measured at six different frequencies (1, 5, 50, 250, 500, and 1000 kHz) using an eight-polar tactile electrode (Inbody720, Biospace, West Des Moines, IA, USA).

### 2.4. Blood Collection and Sample Processing

Venous blood samples were collected in the morning (between 7:00 and 10:00 a.m.) after an overnight fast of at least 8 h and a minimum of 24 h after the last exercise session. Blood was collected into serum tubes, processed according to standard laboratory procedures, centrifuged, aliquoted, and stored at −80 °C until biochemical analyses were performed.

### 2.5. Exercise Protocol

The training program lasted 12 weeks and consisted of Nordic Walking for older participants (age ≥ 60) and a combination of walking and running for people aged 35–50. Both included 36 training sessions (three times a week) divided into three microcycles (6:24:6 training sessions). Each training session was performed in the following order: 10 min warm-up, 45–55 min of endurance training, and 10 min cool-down. In addition, strength exercises, primarily targeting the back muscles, were mandatory for all participants. Only participants with at least 90% attendance were considered to have completed the intervention.

In the first two weeks, participants were introduced to the fundamentals of the training method (continuous and repeated), ensuring they understood the correct technique of the program’s principles. This was particularly important among the older participants.

Starting in the third week, the core training phase involved two primary methods. The first used a repetitive approach with 1000 m segments, while the second used a continuous method aimed at building endurance. A baseline walking test was performed before the intervention to assess participants’ initial walking performance and to allocate them to subgroups with similar walking pace. Training intensity gradually increased over time and was based on the percentage of maximal heart rate (HRmax) determined during the walking test, from 50–60% HRmax at baseline (first 6 units) to 60–70% HRmax (24 units), and in the last six weeks, the intensity of training was maintained with a temporary increase of up to 80–90% HRmax (last 6 units). To monitor the intensity of the exercise, Polar (Finland), with a built-in GPS and an additional HR sensor, was used.

During the eleventh week, outdoor games and activities were introduced to diversify the training experience. The progressive structure of this program ensured a systematic adaptation to training, incorporating technique mastery, endurance training, and a final competitive challenge.

### 2.6. Time-Restricted Eating

All participants were informed about the general rules of the time-restricted eating (TRE) regimen, which involves a 10 h eating window each day, followed by 14 h of fasting. Participants logged their daily eating hours using tables. It was recommended to record the exact start and end times of their daily eating periods. These schedules were monitored after 12 weeks of this study. Only participants with at least 90% adherence to the 14 h fasting protocol were considered to have completed the intervention. Participants were instructed to maintain their usual dietary habits and hydration throughout this study while following the TRE protocol. They were specifically asked not to modify the type, amount, or caloric content of their diet. No recommendations regarding macronutrient composition, total energy intake, or dietary tryptophan intake were provided, as the intervention was designed to modify only the timing of food consumption.

### 2.7. Quantification of Kynurenine Pathway Metabolites

An isotopic dilution method was used with high-performance liquid chromatography coupled to tandem mass spectrometry (LC-MS/MS) technique to determine kynurenine pathway metabolites. The primary circulating metabolites are analyzed, including KYN, KYNA, 3-HK, 3-HAA, PA, QUIN, AA and XANA. A combination of protein precipitation and derivatisation was used to prepare the serum sample (50 µL). Protein precipitation was performed in polypropylene 96-deep-well plates using 250 µL acetonitrile solution, including isotopically labeled internal standards (2H4-L-KYN, 13C2,15N-3-HK, 2H5-KYNA, 2H4-PA, 13C315N-QUIN, 2H4-XANA, 2H3-3-HAA, 13C6-AA) and carried out for 30 min at room temperature at shaking speeds of 900 rcf. After centrifugation, the supernatant (50 µL) was transferred into a fresh 96-well plate and evaporated in a stream of nitrogen (12 L/min, 15 min at 55 °C). Derivatization (n-butylation) reaction was performed by adding n-butanol in 3N hydrochloric acid (25 µL) to the dry residue, and the reaction was carried out for 25 min (60 °C, 450 rpm). After re-evaporation, the dry residue was dissolved in 100 µL of 0.1% formic acid and analyzed using LC-MS/MS.

Chromatographic separation of kynurenine metabolites was carried out using a thermostated column (40 °C) in reverse-phase mode (Agilent, Zorbax Eclipse XDB-C18 Santa Clara, CA 95051 USA, 80 Å, 4.6 × 50 mm, 1.8 µm) at a flow rate of 0.8 mL/min in a linear gradient (from 85%:15% to 3%:97%) of water and acetonitrile, both with 0.1% formic acid as mobile phases. The analysis was performed on a Shimadzu NexeraXR LC-20AD XR liquid chromatograph (Japan, Kyoto) using a CTC PAL-xt autosampler and coupled to a Sciex QTRAP5500+ tandem mass spectrometer equipped with an electrospray ion source (TurboV Ion Source). Ions were observed in positive ion mode at 650 °C with electrospray generated at 4500 V, nebulizer, and drying gas flows of 50 and 60 psi, respectively. Unique fragmentation reactions were observed in single reaction monitoring (SRM, Q1 mass/Q3 mass) mode for both analytes (KYN, 265/136; KYNA, 246/144, 3-HK, 281/152; QUIN, 224/106; PA, 180/106; XANA, 262/160; 3-HAA,154/108; AA, 138/92) and internal standards (2H4-L-KYN 269/140, 13C2, 15N-3-HK3-HK, 284/152; 2H5-KYNA, 251/149; 2H4-PA, 184/110; 13C315N-QUIN, 229/110; 2H4-XANA, 266/164; 2H3-3-HAA, 157/83; 13C6-AA, 144/98) with optimized ion transmission parameters including declustering potential (DP) and collision energy (CE).

Quantitative analysis by isotopic dilution was performed using a 7-point calibration curve covering different working ranges for each analyte: 10–1000 ng/mL for KYN; 1–100 ng/mL for 3-HAA, 3-HK, and KYNA; 5–500 ng/mL for QUIN; 0.5–50 ng/mL for PA and XANA. The Analyst^®^ software 1.6 was used to collect raw data, and MultiQuant^®^ (Sciex, Framingham, MA, USA) was used to process and quantify it.

Study samples were analyzed as single measurements. Analytical precision of the LC–MS/MS method was established during validation using serum samples at three concentration levels, analyzed in five replicates within-run and between days. Within-run CVs ranged from 1.29% to 7.06%, and between-day CVs ranged from 2.35% to 16.93%; the highest between-day CV was observed for anthranilic acid, while CVs for all other metabolites were ≤9.14%.

Tryptophan concentration was measured according to a previously published protocol [23].

### 2.8. Psychological Research Tools Used in This Study

The UWIST Mood Adjective Checklist [27], the original version by Matthews, Chamberlain, and Jones, and the Polish adaptation by Gorynska (2005, UMACL), were used for psychological assessment [27,28]. The test was used to assess mood along the three dimensions of the indigenous affective space: energetic arousal (EA: energized to tired), tense arousal (TA: tense to relaxed), and hedonic tone (HT: happy to sad). The test consisted of 29 adjectives. The respondents were asked to rate each adjective on a four-level scale (from “definitely yes” to “definitely no”) to describe their current mood. The internal reliability scores in the general Polish population were satisfactory—Cronbach’s Alpha for hedonic tone was 0.89, tense arousal was 0.83, and energetic arousal was 0.78. The State-Trait Anxiety Inventory (STAI) by Spielberger [29], in the Polish adaptation by Wrześniewski et al. 2011 [30], was used to assess anxiety as both a transient emotional state (state anxiety) and a relatively stable personality characteristic (trait anxiety). STAI consisted of two subscales: X1 was used to measure state anxiety, and X2 trait anxiety. Each subscale consisted of 20 items, which the test person answered by selecting one of four categorized answers. The internal reliability in the general Polish population of both scales was high—Cronbach’s Alpha for state anxiety was 0.84, and for trait anxiety was 0.94.

### 2.9. Inflammatory Biomarkers Assessment

The concentrations of designated inflammatory biomarkers, including interleukin-6 receptor (IL-6R), tumor necrosis factor-alpha (TNF-α), soluble glycoprotein 130 (sgp130), and tumor necrosis factor receptor superfamily member 1A (TNFRSF1A), were quantified in venous blood serum samples obtained from participants in the morning, following an overnight fast, both prior to and following the 12-week intervention.

Analyses were conducted using Luminex^®^ xMAP^®^ technology on the MAGPIX^®^ platform with a customized Human Magnetic Luminex^®^ Assay panel (R&D Systems, Minneapolis, MN, USA). All procedures were performed according to the manufacturer’s instructions. The Luminex system enables the simultaneous measurement of multiple analytes in a single sample using color-coded magnetic microspheres detected by laser. All samples were determined based on standard curves generated from known calibrators. Sensitivity for the individual parameters was as follows: TNF-α—1.2 pg/mL, sgp130—11.2 pg/mL, TNFRSF1A—41.0 pg/mL, IL-6Rα—0.6 pg/mL. In addition, the Systemic Inflammation Index (SII) was calculated for each participant based on complete blood count parameters using the following formula: SII = (platelet count × neutrophil count)/lymphocyte count.

### 2.10. Statistical Analysis

Statistical analysis was performed using Statistica 13.1 software. Graphs were created in GraphPrism 7 software. All values are expressed as the mean ± standard deviation (SD). The Shapiro–Wilk test was used to assess the normality of data distribution. The Brown–Forsythe test was used to evaluate the homogeneity of variance. First, pre- and post-intervention changes following the endurance exercise + TRE intervention were analyzed. For comparisons between pre- and post-intervention measurements within the same group, the distribution of the paired differences was assessed for normality. When the paired differences were approximately normally distributed, a paired-samples t-test was used. When the distribution of the paired differences deviated from normality, the non-parametric Wilcoxon signed-rank test was applied.

In the second step, differences in changes over time between the OE-TRE and YE-TRE groups were analyzed using a two-way mixed-design ANOVA, with time (pre- and post-intervention) as the within-subject factor and group (OE-TRE vs. YE-TRE) as the between-subject factor. The time × group interaction was considered the primary indicator of whether changes over time differed between the groups. When significant main or interaction effects were observed, appropriate post hoc pairwise comparisons were performed to identify the source of the observed differences. For variables that did not meet the assumptions of parametric analysis, individual change scores (Δ) were calculated as the difference between post-intervention and pre-intervention values (post−pre). The change scores were then compared between the OE-TRE and YE-TRE groups using the Mann–Whitney U test. The choice of the non-parametric procedure was based on the distribution of the change scores within the groups. The significance level was set at *p* < 0.05.

Because participants were retrospectively grouped according to the observed direction of change in anxiety, this post hoc analysis is susceptible to data-driven selection and potential regression-to-the-mean effects and should therefore be considered exploratory and hypothesis-generating. Associations observed within these outcome-defined subgroups should not be interpreted as evidence of causal or mechanistic relationships.

Analyses were performed according to the per-protocol principle and included only participants who completed the 12-week intervention. Owing to incomplete data among participants who withdrew from this study, an intention-to-treat (ITT) analysis was not feasible.

## 3. Results

### 3.1. Anthropometric Characterization of the Subjects

Significant reductions in body weight (BW), fat mass, body mass index (BMI), and body fat percentage were observed in all participants and within both age groups (*p* < 0.05). In contrast, changes in total body water (TBW), fat-free mass (FFM), and skeletal muscle mass (SMM) were less pronounced, with statistically significant increases observed only in the YE-TRE group (Table 1).

### 3.2. Changes in Physical Performance Among All Participants

Following the 12-week program combining physical training with time-restricted eating, differences between the first and second measurements were observed across several measures of physical performance. Physical fitness was assessed as described by Kortas et al. [31]. Participants significantly increased their left arm curl repetitions from 23.64 ± 6.18 to 28.06 ± 6.15 (*p* < 0.01), and right arm curl repetitions from 23.32 ± 6.11 to 31.11 ± 5.60 (*p* < 0.01). Flexibility also improved, with left back scratch scores increasing from −10.67 ± 10.87 cm to −9.51 ± 10.16 cm (*p* = 0.01), and right back scratch scores from −7.28 ± 10.28 cm to −3.23 ± 8.45 cm (*p* < 0.01). Chair sit-and-reach scores improved on the left side from 2.69 ± 9.94 cm to 4.80 ± 8.57 cm (*p* < 0.01), and on the right side from 2.46 ± 9.72 cm to 4.37 ± 8.35 cm (*p* < 0.01). Agility and balance improved as well, as shown by faster performance in the 8-foot up-and-go test, with completion time decreasing from 5.67 ± 0.96 s to 4.90 ± 0.66 s (*p* < 0.01). Lower body strength increased, as indicated by a rise in chair stand repetitions from 17.47 ± 3.74 to 21.23 ± 3.06 (*p* < 0.01). In contrast, hand grip strength remained unchanged, with no significant difference for the right hand (34.73 ± 10.93 kg to 33.10 ± 12.52 kg, *p* = 0.11) or the left hand (32.94 ± 10.69 kg to 31.29 ± 11.92 kg, *p* = 0.12).

These findings indicate higher performance at the second measurement across several domains of physical fitness.

### 3.3. Tryptophan Metabolism and Inflammation Indicators Regarding Psychological Assessment

Initial analyses were conducted on all 71 participants who underwent TRE combined with exercise (TRE + Ex), without stratification by age (Supplementary Table S1). These analyses evaluated changes in psychological well-being, metabolic parameters, and inflammatory biomarkers in the entire cohort. The pre-specified whole-cohort analysis did not reveal statistically significant or clinically meaningful changes in the assessed outcomes. Given previous evidence of age-related differences in kynurenine metabolism [25], an exploratory post hoc analysis stratified by age was subsequently performed (Figure 2). The age-stratified analysis began with a comparison of baseline characteristics between the OE-TRE (older adults) and YE-TRE (younger participants) groups (Table 2). A statistically significantly higher concentration of QUIN was observed in the OE-TRE group (370.16 ± 130.72 nmol/L) compared to the YE-TRE group (303.14 ± 111.73 nmol/L, *p* = 0.03). A marginally non-significant difference in the AA/3-HAA ratio was noted (*p* = 0.07), with the OE-TRE group (0.95 ± 0.7) showing a tendency toward higher values relative to the YE-TRE group (0.68 ± 0.45). However, statistically significantly higher ratios in the YE-TRE group were observed for KYNA/QUIN (0.15 ± 0.07 vs. 0.12 ± 0.05, *p* = 0.04), PA/QUIN (0.16 ± 0.09 vs. 0.11 ± 0.04, *p* < 0.01), KYNA/3-HK (1.95 ± 2.59 vs. 0.91 ± 0.96, *p* = 0.02), and (XANA + PA)/3-HK (2.24 ± 2.42 vs. 1.15 ± 1.35, *p* = 0.02).

To explore the relationship between changes in kynurenine metabolites and psychological outcomes, participants were stratified post hoc according to the direction of change in anxiety scores between the first and second measurements (Figure 3). Given earlier findings suggesting the relevance of anxiety in the context of lifestyle interventions, changes in anxiety scores were examined as part of an exploratory post hoc analysis [32,33].

Among the 51 participants with complete psychological assessment data in the second measurement, 34 (66.7%) obtained lower scores in anxiety, whereas 17 (33.3%) exhibited either no change or an increase in anxiety. In the subgroup with decreased anxiety, participants also demonstrated higher levels of energetic arousal and hedonic tone, along with lower levels of tense arousal and trait anxiety. In contrast, individuals with higher anxiety showed a less favorable psychological profile, including lower hedonic tone and increased tense arousal. Within this exploratory, anxiety-defined subgroup analysis, a decrease in anxiety co-occurred with a reduction in serum 3-hydroxykynurenine (3-HK) (Figure 4). Conversely, in participants whose anxiety increased or remained unchanged, an increase in serum 3-HK levels was observed. Importantly, this finding was restricted to the post hoc anxiety-defined subgroups and should not be interpreted as evidence of a general reduction in 3-HK in the study cohort.

Moreover, analysis of psychological variables revealed higher energetic arousal (EA) in the older group (33.32 ± 3.98 vs. 31.17 ± 4.4, *p* = 0.04). In the next stage of analysis, differences in longitudinal changes between age groups were assessed between age groups (Table 2). In this exploratory age-stratified analysis, 3-HK did not show a significant change in either age group, and the magnitude of change did not differ between groups (OE-TRE: Δ = −3.21 nmol/L, 95% CI −10.46 to 4.04; YE-TRE: Δ = −5.45 nmol/L, 95% CI −14.27 to 3.36; between-group *p* = 0.58). The OE-TRE group exhibited an increase in AA levels (Δ = 7.85 nmol/L), whereas the YE-TRE group showed a decrease (Δ = −2.62 nmol/L; time × group (TxG) interaction *p* < 0.01). Similar group-dependent patterns of change were observed for the AA/3-HAA ratio (OE-TRE: Δ = 0.33 vs. YE-TRE: Δ = −0.02; TxG interaction *p* = 0.04). In addition, the YE-TRE group demonstrated an increase in tryptophan (TRP) concentration, with a shift of 5.03 µmol, while the OE-TRE group showed a less pronounced increase (Δ = 1.08 µmol; TxG interaction *p* = 0.01). Finally, the KYN/TRP ratio decreased in the YE-TRE group (Δ = −0.03 µmol/µmol), with changes over time differing between the YE-TRE and OE-TRE groups (TxG interaction *p* < 0.01). In the exploratory post hoc subgroup defined by an observed increase in 3-HAA, a concurrent decrease in sgp130 protein was observed (Figure 2A). The (XANA + PA)/3-HK ratio increased in the OE-TRE group (Δ = 1.09), whereas it decreased in the YE-TRE group (Δ = −0.18), with a marginal time × group interaction (*p* = 0.05) (Figure 2B). In contrast, the ln 3-HAA/ln 3-HK ratio was higher at the second measurement than at baseline only in the OE-TRE group (Figure 2C).

The repeated measures ANOVA revealed a significant time × group (TxG) interaction for 3-HK, XANA, and TRP (TxG interaction *p* = 0.02, 0.05, and 0.04, respectively), indicating differential changes between the groups defined according to the direction of change in X1 over the course of the intervention (Supplementary Table S1). Within these exploratory anxiety-defined subgroups, changes in psychological indicators co-occurred with differences in 3-HK levels.

## 4. Discussion

In this study, higher scores in psychological well-being co-occurred with changes in selected kynurenine pathway metabolites during a 12-week program combining endurance exercise with time-restricted eating. Although age-stratified analyses were exploratory and conducted post hoc, they suggest the possibility of differences between younger and older adults, although this study was not designed or powered to evaluate age as a factor associated with differences in changes over time. These observations should also be interpreted in light of the fact that exercise modality differed between age groups, preventing separation of the potential influence of age from differences in training stimulus. Given these observations, it is worth considering whether life stage and related contextual factors may have contributed to the observed differences between groups. Notably, elderly women—who constituted a substantial portion of our sample—are known to have an increased prevalence of mood instability, including depression and anxiety [34]. Psychosocial factors associated with ageing may also contribute to differences in psychological responses and should be considered when interpreting these findings. In this context, exploratory analyses considering age and anxiety may provide hypotheses regarding potential differences in patterns of change across distinct life stages.

Turning to the results, a higher baseline concentration of quinolinic acid (QUIN) was observed in the older adults (OE-TRE) group compared to the younger adults (YE-TRE) group; however, the magnitude of change in QUIN did not differ between the groups over time. Ageing has previously been associated with elevated concentrations of kynurenine (KYN), kynurenic acid (KYNA), and QUIN in both serum and cerebrospinal fluid [25]; however, in our study, no baseline differences were observed in serum KYN or KYNA between age groups. Although QUIN has limited ability to cross the blood–brain barrier under physiological conditions, elevated peripheral levels may still contribute to the formation of reactive oxygen species (ROS) and lipid peroxidation [35], posing potential peripheral risks. There is evidence that anthranilic acid (AA) and 3-hydroxyanthranilic acid (3-HAA) are involved in QUIN formation via interactions with 3-hydroxyanthranilic acid oxidase (3HAO). This enzyme is present in central neurons, and AA acts as a potent inhibitor, reducing the conversion of 3-HAA to QUIN and picolinic acid (PA). Given that AA can cross the blood–brain barrier, increased serum AA concentrations may also reflect increased central availability, although this was not assessed in the present study. Thus, it is plausible that in healthy individuals, elevated endogenous AA may limit the conversion of 3-HAA to QUIN [36]. These findings may be consistent with a potential compensatory mechanism in older adults, although the present study does not allow conclusions regarding central QUIN formation or neurotoxicity. Additionally, AA has been indirectly associated with anti-inflammatory effects [37], and elevated plasma AA concentrations have been documented in conditions such as cardiovascular disease [38], osteoporosis and migraine [39]. Some authors propose that increases in AA and the AA/3-HAA ratio could represent a protective response to mitigate both primary and secondary tissue damage [36]. Therefore, any interpretation regarding potential neuroprotective or neurotoxic mechanisms should be considered speculative and based on existing experimental evidence, as the present study assessed kynurenine metabolites exclusively in peripheral blood.

In our study, AA increased in the OE-TRE group and showed little change in the YE-TRE group, with the magnitude of change differing significantly between groups (*p* < 0.01). Similarly, the change in the AA/3-HAA ratio differed between the OE-TRE and YE-TRE groups (*p* = 0.04). The increase in AA in the OE-TRE group also co-occurred with lower TNF-α levels. Importantly, the magnitude of change in TNF-α differed significantly between the groups (*p* < 0.01), with a reduction observed in the OE-TRE group and little change in the YE-TRE group. Although previous experimental and clinical studies have suggested anti-inflammatory properties of AA, the present study did not formally evaluate associations between changes in AA and TNF-α. Therefore, these observations should be interpreted as co-occurring findings rather than evidence of a mechanistic relationship. Moreover, a study in patients with depressive disorders found that elevated AA levels were inversely correlated with pro-inflammatory MCP-1 and anti-inflammatory IL-1RA [40]. The KYN/TRP ratio, a marker of indoleamine 2,3-dioxygenase (IDO1) activity, is known to increase with age and may serve as an indicator of ‘inflammaging’—the chronic low-grade inflammation [40,41]. In the present study, the change in the KYN/TRP ratio differed significantly between the OE-TRE and YE-TRE groups (*p* < 0.01), with a reduction observed in the YE-TRE group and little change in the OE-TRE group. This finding indicates a difference in the magnitude and direction of change between the groups; however, given the exploratory nature of the age-stratified analysis and the different exercise modalities, it should not be interpreted as evidence that age itself moderated the intervention effect. Psychological assessment revealed higher levels of energetic arousal among older participants, which may reflect greater motivation or engagement in structured lifestyle interventions, although psychosocial factors associated with different life stages may also have contributed [42]. In addition, in a separate exploratory post hoc analysis, participants were stratified according to the direction of change in 3-HAA concentrations during the intervention. Participants whose 3-HAA concentrations increased exhibited a concurrent reduction in sgp130 levels. Because the subgroups were defined according to the observed change in 3-HAA and no formal association analysis between changes in 3-HAA and sgp130 was performed, this finding should be interpreted as exploratory and hypothesis-generating and not as evidence of a mechanistic relationship between kynurenine metabolism and inflammatory signalling.

The increase in the (XANA + PA)/3-HK ratio observed in older participants reflects a relative change in the circulating kynurenine metabolite profile. However, because only peripheral blood metabolites were measured, these findings should not be interpreted as evidence of altered central neuroprotection or neurotoxicity. The interpretation of changes in kynurenine pathway metabolites should also consider the potential influence of B-vitamin status. Riboflavin (vitamin B2) and vitamin B6 serve as cofactors for enzymes involved in kynurenine metabolism and may therefore influence the distribution of metabolites within the pathway [38,43,44,45]. However, as the status of these vitamins was not assessed in the present study, their contribution to the observed metabolic changes cannot be determined and should be considered only as a potential modifying factor. The inclusion of anxiety level analysis was justified by evidence suggesting that the kynurenine pathway may play a key role in the pathophysiology of anxiety disorders [34]. In particular, metabolites such as KYN and KYNA have been linked to the regulation of neural excitability, with imbalances reported in individuals with heightened anxiety symptoms [12]. Prior studies suggest that physical activity-based interventions may be associated with changes in anxiety and kynurenine pathway regulation [46]. Based on this, we included anxiety scores as an exploratory endpoint within our broader assessment of psychological well-being. In addition, we considered the potential influence of age and life stage on changes in psychological outcomes. To investigate the relationship between kynurenines and psychological outcomes, participants were stratified post hoc according to the direction of change in anxiety scores between the first and second measurements. This analysis was exploratory, and no causal inference can be drawn from these observations. Within these anxiety-defined subgroups, lower anxiety levels co-occurred with reduced serum 3-HK concentrations, whereas stable or increased anxiety was accompanied by higher 3-HK levels. These findings are consistent with previous reports suggesting that alterations in kynurenine metabolism may accompany changes in psychological well-being. Therefore, fluctuations in serum 3-HK should be interpreted as co-occurring with changes in psychological responses rather than predicting them, and these observations should be regarded as correlational rather than causal. To the best of our knowledge, this is the first study investigating the relationship between exercise, TRE and psychological well-being. Previous studies evaluating TRE alone have reported mixed results. For example, a TRE intervention in 19 individuals with type 2 diabetes mellitus did not result in significant improvements in psychological well-being or quality of life [47]. Similarly, other studies reported no significant changes in stress levels, Beck Depression Inventory, or Beck Anxiety Inventory scores following TRE [32]. In contrast, Inoue et al. (2004) observed reduced anxiety-like behavior in rats subjected to chronic food restriction [33]. A recent systematic review on TRE and health-related quality of life found both significant and non-significant effects across diverse populations, including overweight sedentary adults, obese individuals, and shift workers [48]. Overall, within these exploratory post hoc anxiety-defined subgroups, changes in serum 3-HK co-occurred with changes in mood and anxiety. This observation should not be interpreted as evidence of a general reduction in 3-HK in the study cohort. Moreover, because only peripheral blood markers were measured, these observations should not be interpreted as direct evidence of central nervous system processes.

### Limitations and Future Research

First, several subgroup analyses were conducted post hoc and should therefore be interpreted as exploratory. In particular, participants were stratified according to the direction of change in anxiety scores and, in a separate analysis, according to the direction of change in 3-HAA concentrations. Because these subgroups were defined based on observed outcomes, the resulting findings should be regarded as hypothesis-generating and should not be interpreted as evidence of causal or mechanistic relationships. Psychological analyses were limited to the 51 participants with complete assessment data, which may have introduced selection bias and may limit the generalizability of these findings. Second, the predominance of female participants (69%) may limit the generalizability of the findings to male populations. Third, the observed effects of the intervention cannot be attributed solely to changes in kynurenine pathway metabolites, as other relevant neuroactive compounds, such as endorphins, were not assessed. Moreover, we are aware that mood indices as well as anxiety as a state may be linked to a variety of factors; however, we found the interplay between psychological and biochemical parameters. Finally, the analysis of age-related differences was exploratory and conducted post hoc, with no prior specification in the study protocol. Therefore, these results should be interpreted cautiously due to an increased risk of type I error and limited statistical power to detect interaction effects. This study was structured as a prospective cohort intervention and did not incorporate a non-intervention control group. The lack of a comparator arm constitutes a methodological limitation that hampers the capacity to establish causal relationships and to distinctly separate the effects of time, inherent mood variations, or external factors from the specific impact of the combined TRE and exercise intervention. Although pre- and post-intervention comparisons served as an internal reference, this method does not account for all potential confounding variables. Moreover, only participants who completed the 12-week intervention were included in the final analysis (per-protocol), and an intention-to-treat (ITT) analysis was not possible due to incomplete data among some individuals who withdrew. This may have introduced selection bias and resulted in an overestimation of intervention effects. Because complete baseline data were not available for all participants who withdrew from this study, a formal comparison between completers and non-completers could not be performed. The dietary component of the intervention focused solely on restricting the time window for food intake. Dietary intake, including total energy intake, macronutrient composition, and tryptophan intake, was not monitored or controlled. Therefore, the contribution of dietary differences, including variations in tryptophan intake and other nutritional factors known to influence kynurenine metabolism, cannot be excluded. Although adherence to the TRE protocol was monitored, the specific timing of food intake within the prescribed eating window was not analyzed. Therefore, potential differences in the timing of food intake between participants were not considered in the present analyses. An additional limitation relates to the age-stratified analyses. Because the exercise protocol differed between age groups, with older participants performing Nordic walking and younger participants performing a combination of walking and running, age and exercise modality were inherently confounded. Consequently, the observed differences between OE-TRE and YE-TRE cannot be attributed solely to age and may also reflect differences in exercise mode, mechanical load, training stimulus, baseline fitness, or other age-related factors. Future studies should apply identical exercise protocols across age groups to disentangle the independent contribution of age from exercise modality. Furthermore, we did not evaluate menstrual cycle phase or menopausal status. Because the study population consisted predominantly of women and the older age group likely included a substantial proportion of postmenopausal participants, differences in hormonal status may have influenced mood, inflammatory markers, body composition, and tryptophan metabolism. Consequently, hormonal status may represent an additional source of confounding when interpreting the exploratory age-stratified analyses. Future research should include control or comparison groups, incorporate a wider array of biochemical markers related to mood regulation, and aim for a more balanced sex distribution to enhance generalizability and clarify causal relationships. Moreover, it is worth modifying the instructions for the psychological assessment and asking participants to subjectively compare their results from the first with the second measurement. Because multiple metabolites, metabolite ratios, inflammatory markers, and psychological variables were evaluated, the possibility of type I error resulting from multiple statistical testing cannot be excluded. Therefore, these findings should be regarded as hypothesis-generating and require confirmation in adequately powered prospective studies.

## 5. Conclusions

In conclusion, the present study supports the findings that subjective (psychological) and objective data (metabolic and inflammatory) co-occurred during the 12-week intervention. We are aware that we cannot establish that TRE plus endurance exercise caused these changes, that kynurenine-pathway modulation mediated improvements in mood, or that age modifies the intervention response. However, it is the first study in which such linkage was analyzed. Exploratory analyses further suggested possible age-related differences in kynurenine pathway responses; however, this study was not designed or powered to establish age as a moderator of intervention response. Because of the single-arm pre–post design, these findings should be interpreted as associative and hypothesis-generating rather than causal. Overall, the present findings support an association between psychological well-being and peripheral kynurenine metabolism observed during the intervention, while further controlled studies are needed to clarify the underlying mechanisms and causal relationships.

## Figures and Tables

**Figure 1:**
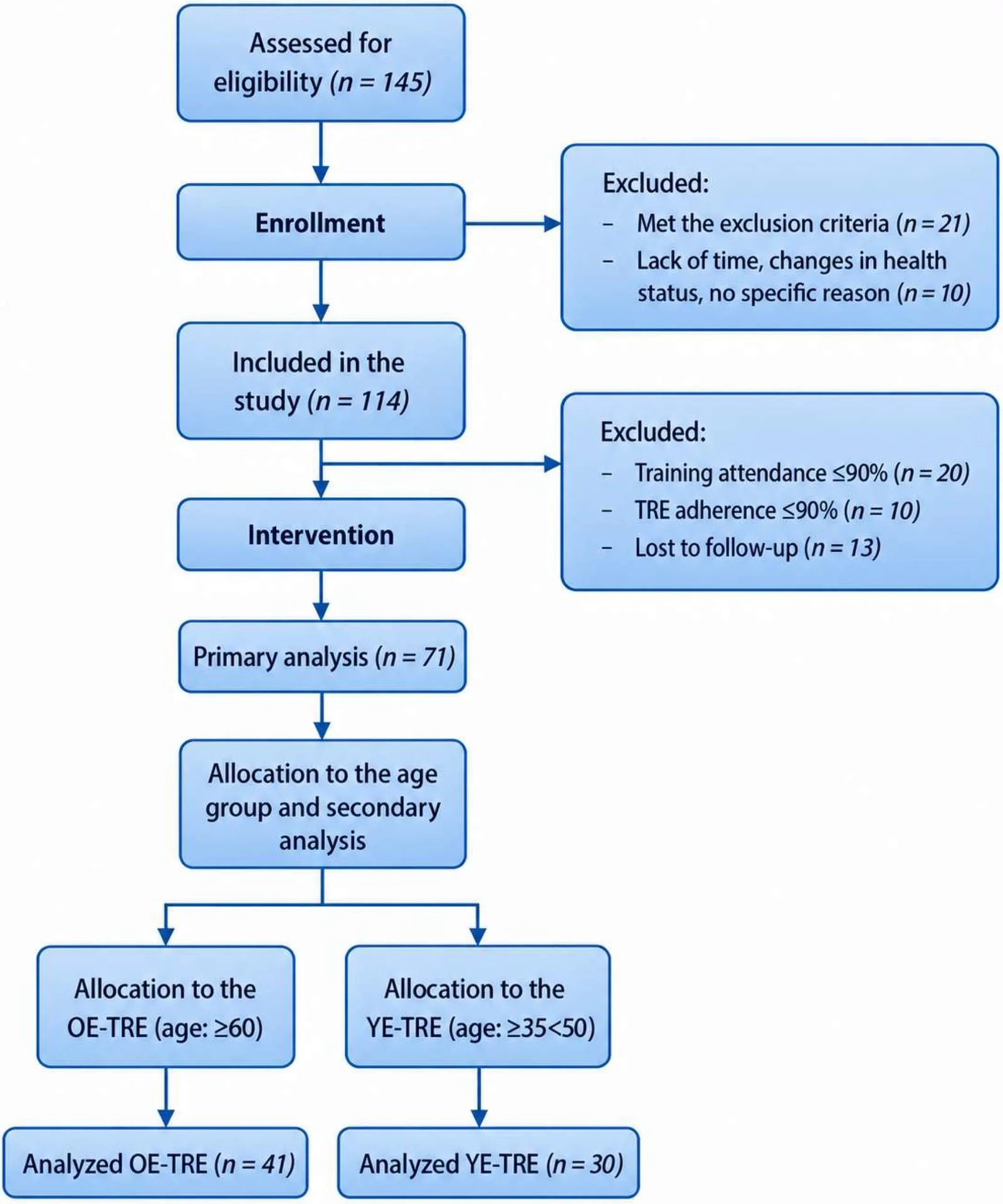
Flow diagram of this study.

**Figure 2:**
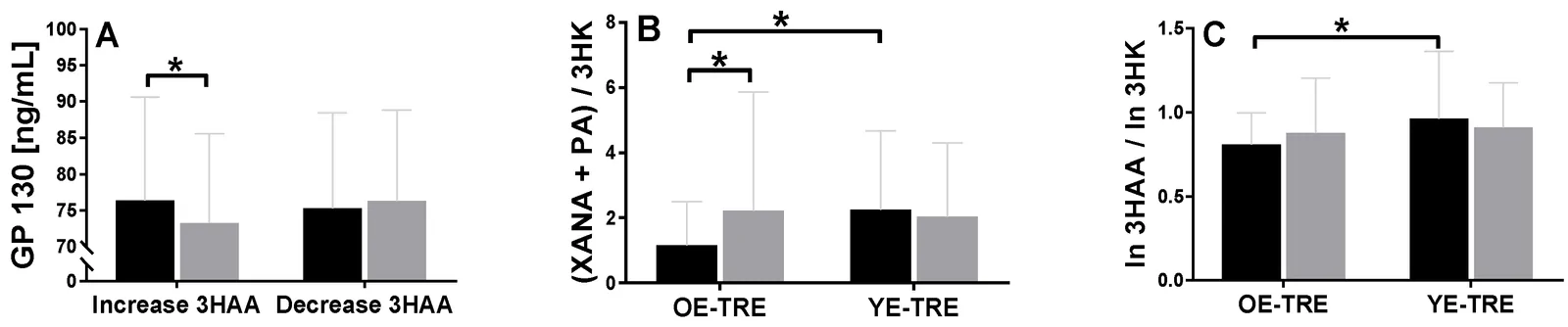
Changes in soluble glycoprotein gp130 concentration and selected tryptophan metabolites observed between the first (black and second measurements grey). (**A**) Changes in GP130 concentration levels among participants with increased and decreased levels of 3-HAA. (**B**) Changes in (XANA + PA)/3-HK levels between pre- and post-intervention in the older adults (OE-TRE) group and the younger adults (YE-TRE) group. (**C**) Changes in ln 3-HAA/ln 3-HK between pre- and post-intervention in OE-TRE and YE-TRE. Bars represent measurements obtained before and after the intervention. GP130—soluble glycoprotein 130, 3-HK—3-Hydroxykynurenine; XANA—Xanthurenic acid; PA—Picolinic acid; * significant difference in soluble gp130 concentration levels between all individuals with increase in 3-HAA before and after intervention (**A**), * significant difference in (XANA + PA)/3-HK ratio in OE-TRE group before and after intervention, as well as significant difference in (XANA + PA)/3-HK ratio between OE-TRE and YE-TRE groups before intervention (**B**), * significant difference in ln 3-HAA (**C**)/ln 3-HK ratio between OE-TRE and YE-TRE individuals before the intervention; *p* ≤ 0.05. Data are mean ± SD.

**Figure 3:**
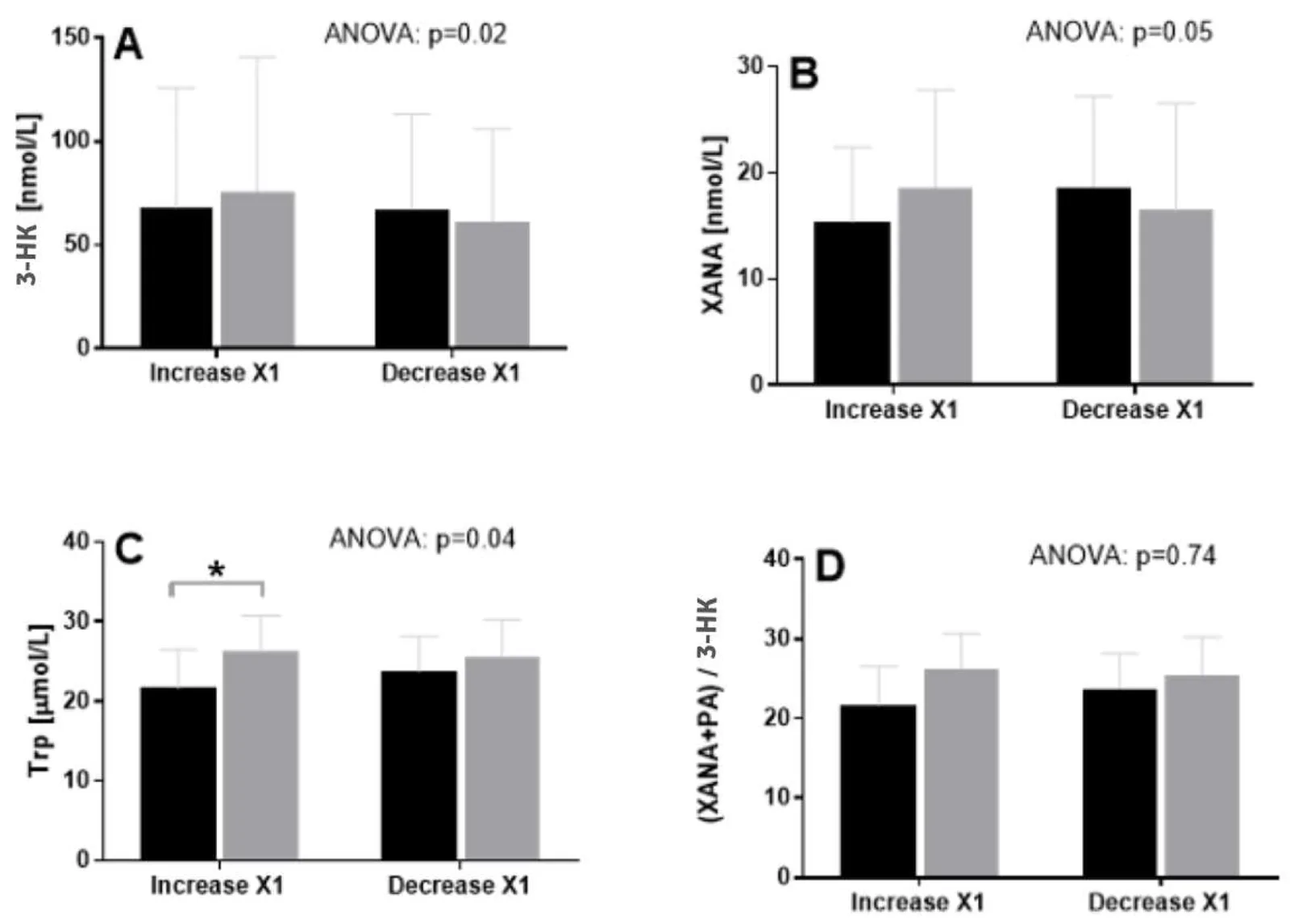
Changes in anxiety level (X1) between the first (black and second measurements grey). Changes in 3-HK (**A**), XANA (**B**), TRP (**C**), and (XANA + PA)/3-HK (**D**) in exploratory post hoc subgroups defined according to the direction of change in anxiety (X1) following the intervention. Bars indicate concentrations before and after the intervention. In participants whose anxiety decreased following the intervention, a decrease in 3-HK (**A**) and XANA (**B**) concentrations was observed. In participants whose anxiety increased or remained unchanged, an increase in TRP (**C**) was observed. NOTE: 3-HK—3-Hydroxykynurenine; XANA—Xanthurenic acid; TRP—Tryptophan; PA—Picolinic acid; * significant difference between pre- and post-intervention in a group with increased X1 (**C**); *p* ≤ 0.05. Data are mean ± SD.

**Figure 4:**
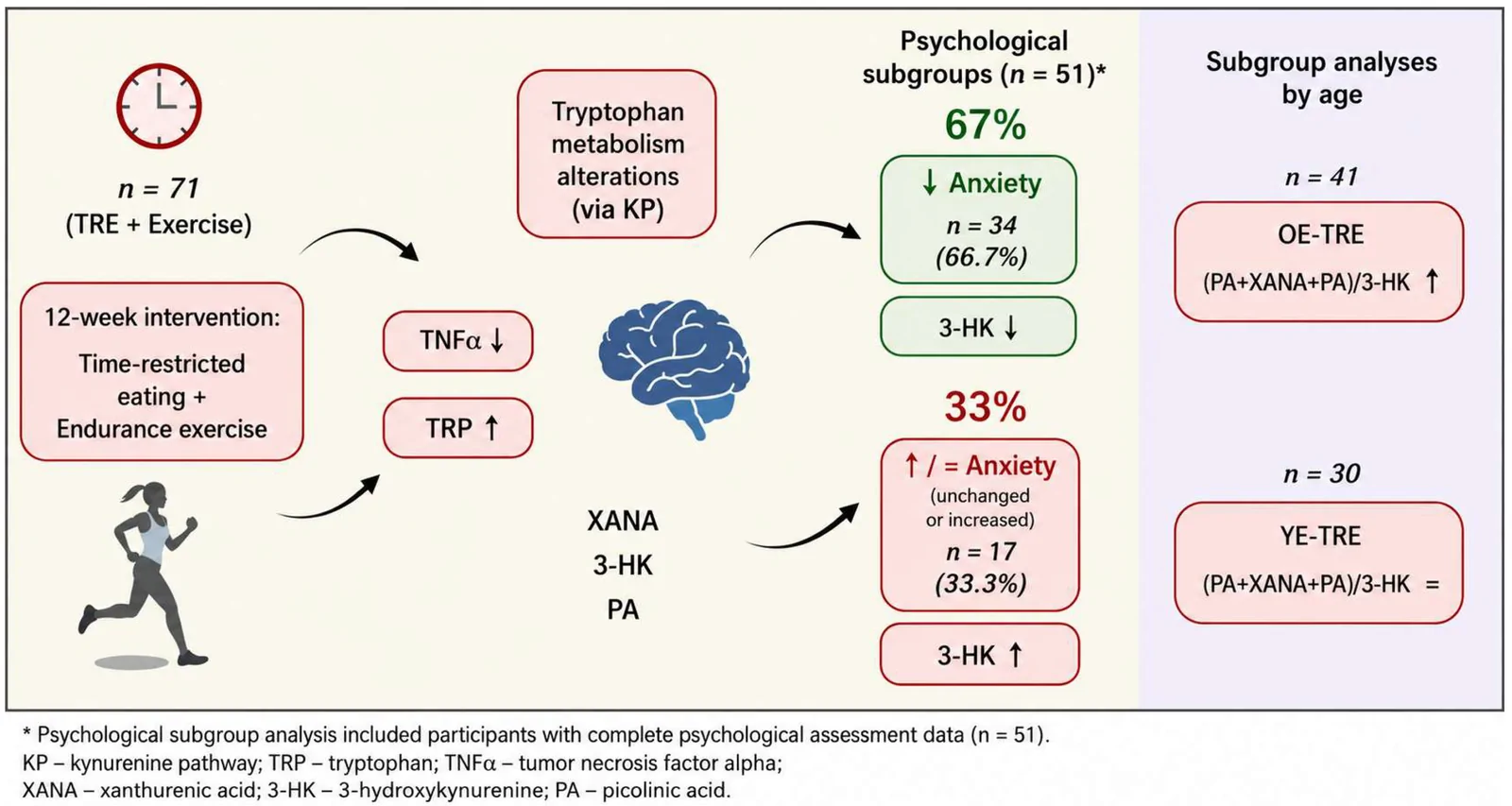
Associations between changes in kynurenine metabolites and changes in psychological well-being during a 12-week program combining endurance exercise and time-restricted eating in OE-TRE and YE-TRE participants. Among the 51 participants with complete psychological assessment data, 34 (66.7%) showed a decrease in anxiety, accompanied by lower serum 3-HK levels, whereas 17 participants (33.3%) exhibited unchanged or increased anxiety together with higher 3-HK levels. An increase in the (XANA + PA)/3-HK ratio was observed in the OE-TRE group, whereas no corresponding change was observed in the YE-TRE group. TRP—Tryptophan; 3-HK—3-Hydroxykynurenine; XANA—Xanthurenic acid; PA—Picolinic acid; TNFα—tumor necrosis factor alpha. This figure was created with BioRender.com.

**Table 1:** Anthropometric characteristics of the all participants before and post-intervention.

	All Participants (*n* = 71, Age ≥ 35)			OE-TRE (*n* = 41, Age ≥ 60)			YE-TRE (*n* = 30, Age ≥ 35 < 50)			
	I	II	*p*	I	II	*p*	I	II	*p*	Between-Group Difference in Change (*p*)
**Age [years]**	58.01 ± 15.82			70.55 ± 6.42			41.84 ± 7.1			
**Gender F/M**	49/22									
**BW [kg]**	77 ± 15.19	75.7 ± 14.52	**<0.01**	74.36 ± 13.64	73.22 ± 12.86	**<0.01**	80.49 ± 16.61	78.91 ± 16.06	**<0.01**	0.97
**Fat [kg]**	25.35 ± 9.34	23.46 ± 9.06	**<0.01**	26.23 ± 8.98	24.56 ± 8.75	**<0.01**	24.18 ± 9.81	22.04 ± 9.4	**<0.01**	0.65
**TBW [kg]**	38.05 ± 7.89	38.35 ± 7.99	0.20	35.62 ± 6.14	35.76 ± 6.2	0.77	41.26 ± 8.84	41.69 ± 8.86	**0.03**	0.06
**FFM [kg]**	51.54 ± 11.05	51.96 ± 11.38	0.13	47.93 ± 8.74	48.15 ± 9.24	0.81	56.31 ± 12.06	56.87 ± 12.13	**0.03**	0.48
**SMM [kg]**	28.67 ± 6.55	28.91 ± 6.62	0.20	26.49 ± 4.99	26.62 ± 5.04	0.88	31.54 ± 7.3	31.85 ± 7.3	**0.04**	0.11
**BMI [kg·m^−2^]**	27.15 ± 4.21	26.61 ± 3.96	**<0.01**	27.28 ± 4.17	26.77 ± 3.95	**<0.01**	26.97 ± 4.32	26.41 ± 4.03	**<0.01**	0.84
**Fat %**	32.06 ± 8.69	30.54 ± 9.13	**<0.01**	33.91 ± 8.31	32.91 ± 8.72	**<0.01**	29.6 ± 8.7	27.5 ± 8.86	**<0.01**	**0.03**
**VFA [cm^2^]**	129.88 ± 36.35	124.87 ± 37.37	**<0.01**	143.9 ± 33.46	141.11 ± 33.98	**<0.01**	111.33 ± 31.8	104.43 ± 31.22	**<0.01**	0.07

Notes: BW, body weight; BMI, body mass index; Fat, fat mass; FFM, free fat mass; TBW, total body water; SMM, skeletal muscle mass; VFA, visceral fat area. Values are means ± SD. I, after overnight fasting, before the intervention; II, after 12 weeks of intervention and overnight fasting. For normally distributed change scores, *p*-values represent the time × group interaction from the two-way mixed-design ANOVA. For non-normally distributed change scores, *p*-values represent the Mann–Whitney U test comparing change scores (post−pre) between the OE-TRE and YE-TRE groups.

**Table 2:** The intervention and tryptophan metabolism, psychological indicators and inflammation markers in subjects—an exploratory analysis by age.

	Baseline Measurements	Mean Change from Baseline (95% CI)				
	OE-TRE (*n* = 41)	YE-TRE (*n* = 30)	*p*	OE-TRE (*n* = 41)	YE-TRE (*n* = 30)	Between-Group Difference in Change (*p*)
**TRYPTOPHAN METABOLITES**						
***3-HK* [nmol/L]**	81.59 ± 46.05	73.45 ± 55.12	0.50	−3.21 (−10.46 to 4.04)	−5.45 (−14.27 to 3.36)	0.58
**KYN [nmol/L]**	3810.9 ± 931.45	3651.17 ± 1039.55	0.50	63.14 (−211.43 to 337.71)	−175.44 (−441.46 to 90.58)	0.22
***KYNA* [nmol/L]**	40.21 ± 16.31	39.97 ± 14.82	0.95	−0.87 (−3.62 to 1.88)	−3.64 (−8.56 to 1.27)	0.75
***QUIN* [nmol/L]**	370.16 ± 130.72	303.14 ± 111.73	**0.03**	−1.45 (−30.22 to 27.32)	−8.6 (−47.21 to 30.01)	0.84
***XANA* [nmol/L]**	17.4 ± 8.64	19.79 ± 9.36	0.27	−0.34 (−2.77 to 2.09)	−1.74 (−5.12 to 1.63)	0.06
***PA* [nmol/L]**	37.82 ± 11.54	44.67 ± 22.59	0.10	4.76 (−1.17 to 10.69)	−0.1 (−7.74 to 7.54)	0.84
***3HAA* [nmol/L]**	27.4 ± 10.44	27.69 ± 9.47	0.91	−0.03 (−3.42 to 3.36)	−0.22 (−3.45 to 3.01)	0.28
**AA [nmol/L]**	23.91 ± 16.23	17.82 ± 12.57	0.09	7.85 (2.31 to 13.38)	−2.62 (−5.49 to 0.25)	**<0.01**
**TRP [µmol/L]**	24.09 ± 4.39	23.87 ± 5.21	0.84	1.08 (−0.94 to 3.09)	5.03 (3.16 to 6.91)	**0.01**
**KYN/TRP**	0.16 ± 0.04	0.16 ± 0.04	0.55	0 (−0.02 to 0.02)	−0.03 (−0.05 to −0.02)	**<0.01**
**KYNA/KYN**	11.15 ± 5.2	12 ± 5.97	0.52	−0.29 (−1.16 to 0.59)	−0.94 (−2.54 to 0.67)	0.44
***QUIN/KYN***	98.42 ± 27.51	87.76 ± 36.64	0.17	−2.36 (−9.65 to 4.92)	1.62 (−8.98 to 12.22)	0.76
**KYNA/QUIN**	0.12 ± 0.05	0.15 ± 0.07	**0.04**	0 (−0.01 to 0.01)	−0.01 (−0.04 to 0.01)	0.26
***PA/QUIN***	0.11 ± 0.04	0.16 ± 0.09	**<0.01**	0.02 (−0.01 to 0.05)	0 (−0.03 to 0.02)	0.93
***KYNA/3-HK***	0.91 ± 0.96	1.95 ± 2.59	**0.02**	0.57 (0.02 to 1.11)	−0.61 (−1.34 to 0.11)	0.11
**AA/3HAA**	0.95 ± 0.7	0.68 ± 0.45	0.07	0.33 (0.05 to 0.6)	−0.02 (−0.19 to 0.15)	**0.04**
**INFLAMMATION MARKERS**						
**SII [cells/L]**	440.16 ± 217.95	483.51 ± 239.33	0.39	−29.18 (−80.06 to 21.70)	−0.38 (−69.91 to 69.14)	0.99
**IL-6RA [ng/mL]**	33.36 ± 6.16	34.48 ± 6.77	0.51	−0.43 (−1.29 to 0.43)	0.42 (−0.54 to 1.39)	0.18
**TNF alpha [pg/mL]**	5.78 ± 1.82	3.94 ± 1.54	**<0.01**	−0.66 (−1.04 to −0.27)	0.12 (−0.1 to 0.34)	**<0.01**
**TNF RI [pg/mL]**	1627.9 ± 382.42	1449.65 ± 281.58	**0.04**	−72.15 (−218.71 to 74.41)	−11.86 (−103.6 to 79.88)	0.49
**MOOD INDICES**						
	***n* = 30**	***n* = 21**		***n* = 30**	***n* = 21**	
**EA [points]**	33.32 ± 3.98	31.17 ± 4.4	**0.04**	0.47 (−0.64 to 1.59)	1.13 (−2.28 to 4.53)	0.63
**TA [points]**	15.15 ± 4.25	16.63 ± 3.87	0.12	−0.65 (−2.14 to 0.84)	−1.19 (−3.8 to 1.42)	0.70
**HT [points]**	33.85 ± 4.18	33.13 ± 3.72	0.46	0.58 (−0.43 to 1.58)	0.88 (−2.14 to 3.89)	0.80
**ANXIETY INDICES**						
	***n* = 30**	***n* = 21**		***n* = 30**	***n* = 21**	
**X1 [points]**	34.85 ± 7.08	35.07 ± 8.2	0.91	−2.2 (−4.32 to −0.08)	0 (−6.44 to 6.44)	0.39
**X2 [points]**	39.88 ± 6.88	43.4 ± 8.32	0.09	−0.92 (−2.4 to 0.55)	−0.94 (−5.05 to 3.18)	0.99

NOTE: 3-HK—3-Hydroxykynurenine; KYN—Kynurenine; KYNA—Kynurenic acid; QUIN—Quinolinic acid; XANA—Xanthurenic acid; PA—Picolinic acid; 3-HAA—3-Hydroxyanthranilic acid; AA—Anthranilic acid; TRP—Tryptophan; psychological indices (*n* = 51): EA—energetic arousal; TA—tense arousal; HT—hedonic tone; X1—state anxiety; X2—trait anxiety; SII—systemic inflammation index; IL-6RA—subunit alpha of Interleukin 6 receptors; TNF alpha—tumor necrosis factor; TNF RI—tumor necrosis factor receptor one; *p* ≤ 0.05. Baseline values are presented as mean ± SD. Changes after the intervention are presented as mean change (95% confidence interval). For normally distributed change scores, *p*-values represent the time × group interaction from the two-way mixed-design ANOVA. For non-normally distributed change scores (italics variables), *p*-values represent the Mann–Whitney U test comparing change scores (post−pre) between the OE-TRE and YE-TRE groups.

## Data Availability

All datasets presented in this study are included in the article/Supplementary Materials.

## Supplementary Materials

The following supporting information can be downloaded at https://www.mdpi.com/article/10.3390/nu18193116/s1, Table S1: Pre- and post-intervention changes stratified according to the direction of change in anxiety level (X1).

## Abbreviations

AA	Anthranilic acid
EA	Energetic arousal
HT	Hedonic tone
3-HK	3-Hydroxykynurenine
3-HAA	3-Hydroxyanthranilic acid
IDO	Indoleamine 2,3-dioxygenase
KAT	Kynurenine aminotransferase
KYN	Kynurenine
KYNA	Kynurenic acid
LC–MS/MS	Liquid chromatography with tandem mass spectrometry
PA	Picolinic acid
QUIN	Quinolinic acid
STAI	State-Trait Anxiety Inventory
TA	Tense arousal
TRE	time-restricted eating
TRP	Tryptophan
UWIST	Mood Adjective Checklist
XANA	Xanthurenic acid
