# Supplementary material for: Changes in Mood and Tryptophan Metabolism Following a 12-Week Endurance Exercise Combined with Time-Restricted Eating in Healthy Adults
Source: Nutrients. 2026 Sep 22;18(19):3116. doi: 10.3390/nu18193116 (PMC13666859; doi:10.3390/nu18193116)
Supplement: Supplementary file 1 [file nutrients-18-03116-s001.zip › nutrients-4480052-supplementary.pdf]

Changes in Mood and Tryptophan Metabolism Following a 12-Week Endurance Exercise Combined with Time-Restricted Eating in Healthy Adults

Ulana Juhas, PhD [a§*], Dagmara Budnik Przybylska, PhD [b§], Borkowska Andzelika, PhD [a], Jakub Antoni Kortas, PhD [c], Joanna Reczkowicz, M.A.[a], Ewa Ziemann, PhD [d], Katarzyna Prusik, PhD [e], Damian Flis, PhD [f], Grażyna Gałęzowska, PhD [a,g], Konrad Kowalski, PhD [a], Malgorzata Zychowska, PhD [h], Barbara Kaczorowska-Hać, MD [i] and Jędrzej Antosiewicz, PhD [a,*]

Table S1: Pre- and post-intervention changes stratified according to the direction of change in anxiety level (X1).

	Increase X1 (n=17)		Decrease X1 (n=34)		ANOVA
	I	II	I	II	p
**TRYPTOPHAN METABOLITES**					
**KYN [nmol/L]**	3567.86 ± 1083.49	3815.15 ± 1177.53	3639.1 ± 869.17	3588.97 ± 778.92	0.21
**KYNA [nmol/L]**	41.64 ± 18.93	42.88 ± 13.58	40.54 ± 14.92	36.31 ± 10.16	0.23
**QA [nmol/L]**	368 ± 157.79	369.4 ± 154.08	341.14 ± 107.05	343.82 ± 106.74	0.87
**PA [nmol/L]**	42.28 ± 19.44	49.34 ± 16.94	34.65 ± 10.25	36.86 ± 15.72	0.54
**3-HAA [nmol/L]**	25.15 ± 9.43	28.65 ± 8.74	26.82 ± 9.44	27.19 ± 12.18	0.26
**AA [nmol/L]**	20.37 ± 14.61	22.93 ± 17.8	21.39 ± 17.06	26.79 ± 23.4	0.27
**KYN/Trp [µmol/µmol]**	0.16 ± 0.04	0.15 ± 0.05	0.16 ± 0.04	0.15 ± 0.04	0.51
**KYNA/KYN [nmol/µmol]**	12.81 ± 6.69	12.39 ± 5.61	11.89 ± 5.34	10.86 ± 4.62	0.78
**QA/KYN [nmol/µmol]**	106.04 ± 35.54	98.51 ± 31.68	97.03 ± 32.8	97.76 ± 30.13	0.28
**KYNA/QA [nmol/nmol]**	0.12 ± 0.05	0.13 ± 0.07	0.13 ± 0.07	0.11 ± 0.05	0.09
**PA/QA [nmol/nmol]**	0.13 ± 0.06	0.16 ± 0.1	0.11 ± 0.05	0.12 ± 0.06	0.32
**KYNA/3-HK [nmol/nmol]**	1.81 ± 1.83	1.86 ± 2.06	1.46 ± 2	1.59 ± 2	0.86
**AA/3-HAA [nmol/nmol]**	0.81 ± 0.49	0.81 ± 0.54	0.86 ± 0.74	1.11 ± 0.96	0.09
**INFLAMMATION MARKERS**					
**SII [cells/l]**	524.45 ± 237.40	505.86 ±322.29	400.14 ± 190.62	406.11 ± 198.94	0.61
**GP130 [ng/mL]**	78.80 ± 14.93	74.61 ± 13.29	73.29 ± 12.14	72.57 ± 11.98	0.15
**IL-6RA [ng/mL]**	34.21 ± 8.55	32.95 ± 8.83	32.86 ± 5.77	32.91 ± 6.47	0.11
**TNF alpha [pg/mL]**	5.04 ± 2.1	4.47 ± 1.82	5.37 ± 1.92	5.07 ± 1.7	0.42
**TNF RI [pg/mL]**	1576.63 ± 379.45	1542.13 ± 384.15	1547.15 ± 303.39	1467.47 ± 343.18	0.69
**MOOD INDICES**					

**EA [points]**	32.61 ± 3.65	31.78 ± 3.51	32.54 ± 4.98	34.03 ± 3.78	0.08
**TA [points]**	15.5 ± 3.73	16.67 ± 3.34	15.4 ± 4.58	13.89 ± 3.74*,#	**0.05**
**HT [points]**	33.33 ± 3.76	32.44 ± 4.23	34.03 ± 4.31	35.4 ± 3.77*,#	**0.05**
		**ANXIETY INDICES**			
**X1 [points]**	31.83 ± 7.1	39.39 ± 8.13*	36.83 ± 7.77	30.43 ± 6.81*,#	**<0.01**
**X2 [points]**	40.28 ± 7.04	42.28 ± 8.7	41.34 ± 7.59	38.97 ± 6.28*	**0.01**

NOTE: 3-HK - 3-Hydroxykynurenine; KYN – Kynurenine; KYNA - Kynurenic acid; QA – Quinolinic acid; XANA – Xanthurenic acid; PA – Picolinic acid; 3-HAA – 3-Hydroxyanthranilic acid; AA – Anthranilic acid; Trp – Tryptophan; psychological indices (n=51): EA – energetic arousal; TA – tense arousal; HT – hedonic tone; X1 – state anxiety; X2 – trait anxiety; SII – systemic inflammation index; GP130 – Glycoprotein 130; IL-6RA – subunit alpha of Interleukin 6 receptors; TNF alpha – tumor necrosis factor; TNF RI – tumor necrosis factor receptor one ; I, after overnight fasting, before the intervention; II, after 12 weeks of intervention and overnight fasting. *significant difference between pre and post intervention in the group with Increase X1 or Decrease X1. # significant difference between post intervention in the Increase X1 and Decrease X1 gruops. p≤0.05. Values are mean±SD.
